# Changes in catastrophic health expenditure in post-conflict Sierra Leone: an Oaxaca-blinder decomposition analysis

**DOI:** 10.1186/s12939-017-0661-4

**Published:** 2017-09-04

**Authors:** Ijeoma Edoka, Barbara McPake, Tim Ensor, Rogers Amara, Joseph Edem-Hotah

**Affiliations:** 1 PRICELESS SA, Wits School of Public Health, University of the Witwatersrand, Johannesburg, South Africa; 2 Nossal Institute for Global Health, University of Melbourne, Australia and Institute for Global Health and Development, Queen Margaret University Edinburgh, Edinburgh, UK; 3 Institute of Health Sciences, University of Leeds, Leeds, UK; 4 ReBUILD Consortium, College of Medicine and Allied Health Sciences, University of Sierra Leone, Freetown, Sierra Leone

**Keywords:** Catastrophic health expenditure, Sierra Leone, Post-conflict, Conflict

## Abstract

**Background:**

At the end of the eleven-year conflict in Sierra Leone, a wide range of policies were implemented to address both demand- and supply-side constraints within the healthcare system, which had collapsed during the conflict. This study examines the extent to which households’ exposure to financial risks associated with seeking healthcare evolved in post-conflict Sierra Leone.

**Method:**

This study uses the 2003 and 2011 cross-sections of the Sierra Leone Integrated Household Survey to examine changes in catastrophic health expenditure between 2003 and 2011. An Oaxaca-Blinder decomposition approach is used to quantify the extent to which changes in catastrophic health expenditure are attributable to changes in the distribution of determinants (distributional effect) and to changes in the impact of these determinants on the probability of incurring catastrophic health expenditure (coefficient effect).

**Results:**

The incidence of catastrophic health expenditure decreased significantly by 18% from approximately 50% in 2003 t0 32% in 2011. The decomposition analysis shows that this decrease represents net effects attributable to the distributional and coefficient effects of three determinants of catastrophic health expenditure – ill-health, the region in which households reside and the type of health facility used. A decrease in the incidence of ill-health and changes in the regional location of households contributed to a decrease in catastrophic health expenditure. The distributional effect of health facility types observed as an increase in the use of public health facilities, and a decrease in the use of services in facilities owned by non-governmental organizations (NGOs) also contributed to a decrease in the incidence of catastrophic health expenditure. However, the coefficient effect of public health facilities and NGO-owned facilities suggests that substantial exposure to financial risk remained for households utilizing both types of health facilities in 2011.

**Conclusion:**

The findings support the need to continue expanding current demand-side policies in Sierra Leone to reduce the financial risk of exposure to ill health.

## Background

Direct payment made by patients at the point of care (out-of-pocket payment) is the major source of financing health care in low income countries [1]. In addition to deterring access to healthcare services, the risks of incurring catastrophic health expenditure due to out-of-pocket payments are well documented [2–4]. Using two cross-sections (2003 and 2011) of the Sierra Leone Integrated Household Survey (SLIHS), this study investigates changes in the incidence of catastrophic health expenditure in post-conflict Sierra Leone. Following previous studies, for examples [3, 5], catastrophic health expenditure is defined as health expenditure exceeding 10% of household total expenditure. This threshold represents the point above which household living standards are believed to be compromised by out-of-pocket health expenditure either through the diversion of financial resources from food and basic necessities, through the depletion of savings and assets or through the accumulation of debt [6]. Health expenditure shares below the threshold are indicative of households’ ability to smooth consumption when faced with unexpected out-of-pocket payments. However, this is only true for households using healthcare services. Health expenditure share may also fall below this threshold if healthcare is needed but not used due to an inability to pay or other access constraint. Given the economic consequences of health shocks [7, 8], possibly worsened by delaying or not seeking healthcare, the measure of catastrophic health expenditure described in this study only partly captures the true extent of financial risks facing households.

### Review of the literature on the determinants of catastrophic health expenditure

In recent years, the emphasis has shifted from simply estimating the extent of catastrophic health expenditure within populations to understanding socioeconomic factors that explain variations in households’ exposure to healthcare financial risks. Studies using household-level data have consistently shown strong correlations between a range of household socio-demographic characteristics and the incidence of catastrophic health expenditure. For example, the incidence of catastrophic health expenditure is correlated with health care need – households with a larger proportion of elderly members or children under the age of five are more likely to incur catastrophic health expenditure [9–12]. Household location (rural vs. urban), as well as types of healthcare service used (inpatient vs. outpatient care) and type of health facility (private vs. public) are correlated with the risk of incurring catastrophic health expenditure [9–11, 13, 14]. Depending on the design features (for example, levels of co-payments, types of benefit package and payment-provider mechanisms), enrolment into health insurance schemes has been shown to either increase protection against catastrophic health expenditure [9, 14, 15], to have a limited effect or to increase the risk of incurring catastrophic health expenditure [10–12, 16, 17].

These studies provide insights into factors that protect households or increase their vulnerability to catastrophic health expenditure and are useful in informing targeted policy decisions. Therefore, in addition to estimating trends in the incidence of catastrophic health expenditure, this paper quantifies the extent to which temporal changes in the determinants of catastrophic health expenditure, including household characteristics and healthcare-seeking behaviours, contributed to changes in the incidence of catastrophic health expenditure in Sierra Leone. Temporal changes in the incidence of catastrophic health expenditure can occur either through a change in the distribution of determinants or through a change in the impact of these determinants. The Oaxaca-Blinder decomposition approach [18, 19] is applied to disentangle these two effects into a distributional effect component, which describes changes attributable to differences in the distribution of characteristics and a coefficient effect component, which describes changes attributable to differences in the impact of these characteristics. Each effect is estimated while holding the other constant.

### Health financing policies in post-conflict Sierra Leone

Historically, Sierra Leone has ranked as one of the poorest countries in the world, an economic situation made worse by the eleven-year (1991–2002) conflict. The conflict was characterized by extensive destruction of life and property, a contraction of the economy and the collapse of public infrastructure across the country. Approximately 2 million people were displaced during the conflict and at the end of the conflict in 2002, an estimated 50,000 people had been killed out of a population of 4.4 million [20, 21].

Although Sierra Leone still lags behind other sub-Saharan African countries in terms of its health and economic indicators [22], some progress has been made since 2002 to rebuild the economy following the end of the conflict [23]. Between 2003 and 2011, the economy grew by 2.5% while the proportion of households living below the poverty line fell by approximately 13%, from 66.4% in 2003 to 52.9% in 2011 [24]. These gains have been felt more in rural areas and in the Northern and Eastern regions where the conflict had been most intense [24].

In the aftermath of the conflict, several policies were implemented to strengthen the health system. In order to increase responsiveness to local needs in pre-conflict marginalized areas, a Local Government Act was passed in 2004 to decentralize some central government functions (including healthcare provision) to local councils [25, 26]. This contributed to the widespread reconstruction and refurbishment of public healthcare facilities nationwide and an increase in access to healthcare services [25].

In 2006, a cost-recovery scheme was reintroduced following a failed attempt to fully implement one in 2002 [27]. The aim of the scheme was to generate revenue within public health facilities and ensure uninterrupted supply of pharmaceuticals and medical supplies, thus minimizing disruptions to the provision of healthcare services.

In 2010, the Government of Sierra Leone launched a Free Healthcare Initiative (FHCI) aimed at increasing access to health services among vulnerable populations [28]. Under the FHCI, user fees were waived in public health facilities for children under the age of five years, pregnant women and mothers of young babies (‘lactating’). In addition, wider health sector reforms were implemented alongside the launch of the FHCI in 2010 to strengthen supply-side functions of the health system. These included salary uplift to motivate healthcare workers, as well as fast-track recruitment and deployment of health workers to underserved areas. Other supply-side reforms where aimed at strengthening the procurement and supply chain management system to ensure uninterrupted supply of medical supplies and equipment to healthcare facilities [28, 29].

These reforms are likely to have important implications for households’ healthcare-seeking behaviours and exposure to healthcare financial risks. For example, the reintroduction of the cost-recovery scheme and user charges in public health facilities can have an effect on households through two possible routes. First, the fall in the use of public healthcare services by some users as a result of higher charges [30, 31] reduces catastrophic spending at the cost of impairing access. Second, for remaining users, the higher charges increase the likelihood of catastrophic health expenditure. The decomposition approach captures both effects, the first as a distributional effect since post cost-recovery, the use of facilities changes and the second as a coefficient effect due to the change in the impact of facility-use on catastrophic health expenditure.

Therefore this paper offers an advantage over previous studies by providing insights not only into the association between household characteristics and the incidence of catastrophic health expenditure but also into the extent to which wider healthcare sector reforms, implemented during the study period and affecting household healthcare-seeking behaviours, may have changed households’ susceptibility to healthcare financial risks.

The rest of the paper is organized as follows: section 2 outlines the SLIHS, study variables and the Oaxaca-Blinder decomposition approach; the results are presented and discussed in sections 3 and 4 respectively; and the final section concludes the paper.

## Methods

### Data

#### Sierra Leone integrated household survey (SLIHS)

The SLIHS is a cross-sectional national representative sample of households in Sierra Leone conducted in 2003 and 2011 to track changes in household living standards and wellbeing. In both surveys, households were selected using comparable sampling strategies. Sierra Leone is divided into four provinces and 14 districts. Each district is in turn sub-divided into lower administrative units: first into local councils (19), then chiefdoms (149) and sections (1322). Each section is further sub-divided into area units known as enumeration areas (EAs), which formed the primary sampling unit of the SLIHS. Households were selected from a sample of EAs in a two stage-sampling process: first, EAs were stratified into rural and urban areas and a random sample of EAs selected to ensure a representative number of households from urban and rural areas. A random sample of households was then drawn from selected EAs.

In 2011, approximately 6800 households were selected. However, due to financial and human resource constraints, a significantly smaller sample of households (approximately 3700) was interviewed in the 2003 survey.

#### Health expenditure

In both years, data on health expenditure were collected using a health questionnaire completed by heads of households on behalf of all household members. The health questionnaire collected data on the use of in- and outpatient health services from both formal and informal service providers as well as expenditure incurred in using these services. Heads of households were asked to report on the use of healthcare services by any household member in a two- or four-week period^1^ (recall periods) prior to the interview date, and any out-of-pocket expenditure incurred as a result. Both years varied in some components of health expenditure and recall periods over which data were collected – a summary is provided in Table 1. For example, in 2011, for inpatient services (hospital charges, medicines and medical supplies), heads of households were asked to report on utilisation of any service 4 weeks prior to the interview date and out-of-pocket expenditure incurred as a result. However, in 2003, household heads reported on inpatient service-use in the 2 weeks preceding the interview date. For consistency, 4-week expenditure data were scaled down to a 2-week period (by dividing health expenditure by 2). In this study, total health expenditure is estimated as the sum of payments made for outpatient consultation and prescription charges, transportation expenditure, hospital charges as well as for medicines (including over-the-counter medicines) and medical supplies (Table 1).

**Table 1:** Household total expenditure components

Household Expenditure	Recall Period	
	2003	2011
Health Expenditure	2 weeks	2 weeks
• Outpatient services:		
Consultation charges; transportation fee; prescription charges^*^		
• Inpatient services:	2 weeks	4 weeks
Consultation and admission charges		
• Medicines & medical supplies, including over-the-counter medications	2 weeks	4 weeks
Food Expenditure	3–12 months	25 days^**^
Non-food Expenditure		
• Frequent purchases	3–12 months	25 days ^**^
• Infrequent purchases	12 months	12 months

*Prescription charges collected only in 2011

^**^Collected in 5-day intervals

#### Food and non-food expenditure

In both years, detailed information was collected on a wide range of food and non-food goods purchased by households. In 2003, household heads were asked to report on food and frequently purchased non-food goods bought in the 3–12 months period prior to the interview date. To limit recall bias, a different approach was adopted in 2011 to collect data for food and frequently purchased non-food goods. Each household received five visits at regular intervals over a one-month period. The monthly food and non-food expenditure for 2011 was estimated as the sum of all purchases made over the entire month.

For infrequently purchased non-food goods and services, expenditure data were collected over a 12-month recall period in both 2003 and 2011 (Table 1). For comparability with health expenditure data, all food and non-food expenditure was scaled down to a 2-week period by dividing by the proportionate number of weeks.

The 2003 and 2011 surveys varied in the types of food and non-food goods included in the survey questionnaire – some goods were included in the 2003 survey questionnaire, but not in the 2011 survey, and vice versa. To allow for comparability across time, only food and non-food goods included in both years are used in estimating total household expenditure. Total household expenditure in each year is estimated as the sum of health, food and non-food goods and services purchased by each household. This is converted to United States Dollars (USD) using purchasing power parity exchange rates^2^ and expressed in 2005 prices.

#### Catastrophic health expenditure

Catastrophic health expenditure is defined as health expenditure exceeding 10% of household total expenditure. First, health expenditure share of total expenditure is estimated for each household and then a binary variable is generated which equals one, when health expenditure share of total expenditure exceeds 10%.

#### Study covariates

This study makes use of variables shown in previous studies to explain variations in households’ exposure to healthcare financial risks. These include household size, proportion of household members below the age of 5 years and above the age of 65 years, household location (rural/urban and West/East/South/North regions) and proportion of unemployed adult household members. Head of household characteristics include age (and age squared), gender, marital status (married/ single, divorced, separated, widowed), religion (Muslim/Christian, other religion, no religion) and education (no education to junior secondary education/some senior secondary education and above).

In addition to household demographic characteristics, this study uses other previously identified determinants of catastrophic health expenditure. These include the proportion of household members reporting ill health as well as the proportion of household members utilising inpatient and outpatient healthcare services. To investigate differential effects of health facility-type, health service utilisation is disaggregated by type of service. This is identified using a combination of two variables available in SLIHS – the owner of the healthcare facility visited (public or private) and the health worker type consulted. For outpatient health care use, this includes use of informal healthcare services (i.e. visits to traditional healers, spiritualists or traditional birth attendants^3^), use of formal public healthcare services (visits to doctors, dentists, pharmacists, nurses, midwives and medical assistants in government-owned facilities) and use of formal private healthcare services (visits to doctors, dentists, pharmacists, nurses, midwives and medical assistants in privately-owned facilities). Formal private healthcare-use is further disaggregated by privately-owned facilities and facilities owned by non-governmental organizations (NGOs) and missionaries. Given that total health expenditure is estimated at the household level, the proportion of household members utilizing each health facility-type is defined as the number of household members utilizing a facility-type divided by the household size.

In 2011, information on type of privately-owned inpatient health facility was aggregated. Therefore we are unable to distinguish between formal and informal private inpatient healthcare-use. As a result only two categories of inpatient health service-use are included – the proportion of household members hospitalised in government-owned facilities and the proportion of those admitted into privately-owned facilities.

Our final sample consists of 1886 households in 2003 and 2800 households in 2011. These include households with complete data on health expenditure and health service utilisation for all household members as well as complete data on household and head of household characteristics.

### The Oaxaca-blinder decomposition approach

The Oaxaca-Blinder decomposition approach [18, 19] is a mean based decomposition method which allows differences in the outcomes of two mutually exclusive groups to be decomposed into a part attributable to group differences in the distribution (or level) of characteristics (referred to as distributional effect) and a part attributable to group differences in the impact of these characteristics (coefficient effect). The decomposition analysis is performed on the basis of the relationship between an outcome variable and a set of observed characteristics:

1 $$ {C}_Y={X}_Y\ {\beta}_Y+{\varepsilon}_Y\kern0.75em ,\kern3.5em E\left({\varepsilon}_Y\right)=0\kern0.75em Y\ \epsilon\ \left\{2003,2011\right\} $$

where *C* is the incidence of catastrophic health expenditure defined as a binary variable which is equal to one when the share of health expenditure in total household expenditure exceeds 10%; *X,* is a vector of observable household characteristics; *β*, a vector of the slope parameters including the intercept and *ε*, the random error term. Given that *E*(*ε*_*Y*_) = 0 , the change in the incidence of catastrophic health expenditure,${\triangle}_C^{\mu }$, can be written as:

2 $$ {\triangle}_C^{\mu }={C}_{2011}-{C}_{2003}=E\left({X}_{2011}\right)\ast {\beta}_{2011}-E{\left({X}_{2003}\right)}^{\ast }{\beta}_{2003} $$

The decomposition of ${\triangle}_C^{\mu }$ is performed in two steps. In the first instance, an aggregate decomposition analysis is performed to identify total distributional and total coefficient effects using the three-fold decomposition approach [32, 33]:

3 $$ {\hat{\Delta}}_C^{\mu }=\underset{{\hat{\Delta}}_{\beta}^{\mu }}{\underbrace{{\overline{X}}_{2003}\ \left({\hat{\beta}}_{2011}-{\hat{\beta}}_{2003}\right)}}+\underset{{\hat{\Delta}}_X^{\mu }}{\underbrace{\left({\overline{X}}_{2011}-{\overline{X}}_{2003}\right){\hat{\beta}}_{2003}}}\kern0.5em +\underset{{\hat{\Delta}}_I^{\mu }}{\underbrace{\left({\overline{X}}_{2011}-{\overline{X}}_{2003}\right)\left({\hat{\beta}}_{2011}-{\hat{\beta}}_{2003}\right)}} $$

where ${\overline{X}}_{2011}$ and ${\overline{X}}_{2003}$ are the 2011 and 2003 covariate means respectively and ${\widehat{\beta}}_H$ and${\widehat{\beta}}_L$ are the corresponding coefficients estimated using a linear probability model. The first term, ${\hat{\Delta}}_{\beta}^{\mu }$, is the total coefficient effect, which represents contributions attributable to group differences in coefficients (including the intercept). The second term, ${\hat{\Delta}}_X^{\mu }$, is the distributional effect, which represents contributions attributable to group differences in the level of characteristics estimated at the mean. The third term, ${\hat{\Delta}}_I^{\mu }$, represents an interaction between group differences in characteristics and coefficients as well as differences in residuals. Given the ambiguity in the interpretation of the interaction term [34], this study focuses only on interpreting the distributional and coefficient effects.

Second, a detailed decomposition is performed to identify the contribution of each covariate to ${\triangle}_C^{\mu }$, both through changes in the level of covariate means and changes in the magnitude of their effects. The detailed decomposition relies on the additive linearity assumption implied by eq. 1. This means that total distributional and total coefficient effects are each the sum of the contribution of individual covariates:

4 $$ {\hat{\Delta}}_X^{\mu }=\sum_{k=1}^K\left({\overline{X}}_{2011,k}-{\overline{X}}_{2003,k}\right){\hat{\beta}}_{2003,k} $$

and

5 $$ {\hat{\Delta}}_{\beta}^{\mu }=\left({\hat{\beta}}_{2011,\kern0.5em 0}-{\hat{\beta}}_{2003,0}\right)+\sum_{k=1}^K\ \left({\hat{\beta}}_{2011k}-{\hat{\beta}}_{2003,k}\right){\overline{X}}_{2003,k} $$

where *k* represents the *k*th covariate and ${\widehat{\beta}}_{2011,0}$ and ${\widehat{\beta}}_{2003,0}$ are the estimated intercept coefficients from 2011 and 2003, respectively. For categorical covariates with more than two categories, the result of the detailed decomposition of the coefficient effect is dependent on the choice of the base or omitted category. Therefore the coefficient effect of each categorical variable is normalised and presented as a combined effect [32, 35].

The decomposition analysis is performed using the STATA user-written command, ‘**oaxaca**’, specifying a linear probability model [36]. While ‘**oaxaca**’ can support a probit or logit model specification, the linear probability model is used given that ‘**oaxaca**’ applies the decomposition to the linear predictions from the model [36]. This means that ‘**oaxaca**’ with a logit or probit model specification will not be expressed on a probability scale but in terms of log odds (in the case of a logit model) or z-scores (in the case probit models), resulting in difficulties interpreting the results of the decomposition analysis.

## Results

### Summary statistics

Table 2 shows the mean distribution of household characteristics and estimates of the incidence of catastrophic health expenditure in 2003 and 2011. The incidence of catastrophic health expenditure, defined at a threshold of 10%, decreased significantly between 2003 and 2011 by approximately 18 percentage points. Household economic well-being improved between the two study years – total household expenditure per capita increased, largely driven by an increase in household food expenditure (Table 2).

**Table 2:** Mean Household Characteristics

	2003	2011	Difference^±^	Full Sample
Health expenditure share of total expenditure > 10% (CHE)	0.501	0.32	−0.181^***^	0.41
Socioeconomic inequality in CHE ^¥^	0.060	0.068^***^	0.008	0.0343
Household Characteristics				
Below median total expenditure	0.555	0.418	−0.137^***^	0.486
Total expenditure /capita (US$)	39.87	97.63	57.76^**^	68.88
Food expenditure/capita (US$)	15.93	45.19	29.26^***^	30.63
Non-food expenditure/capita (US$)	16.74	46.02	29.28	31.45
Health expenditure (US$)	19.92	15.03	−4.89	17.46
Food share of total expenditure	16.64	10.07	−6.56^***^	13.34
Health share of total expenditure	48.27	63.49	15.22^***^	55.91
Household size	5.576	5.378	−0.198^†^	5.477
Log household size	1.588	1.561	−0.027	1.574
Children under 5 years	0.142	0.106	−0.036^***^	0.124
Adults over 65 years	0.045	0.039	−0.005	0.042
Unemployed adults	0.305	0.377	0.072^***^	0.3411
Ill health	0.399	0.191	−0.208^***^	0.295
Household location				
Rural	0.607	0.67	0.063^***^	0.639
Western region	0.188	0.15	−0.038^**^	0.169
Eastern region	0.239	0.284	0.045^***^	0.261
Northern region	0.291	0.418	0.127^***^	0.355
Southern region	0.283	0.148	−0.135^***^	0.215
Health facility				
Formal Public	0.090	0.095	0.005	0.092
Formal NGO & Missionary	0.012	0.004	−0.008^***^	0.008
Formal Private	0.064	0.022	−0.042^***^	0.043
Informal Private	0.010	0.016	0.006^**^	0.013
Public Hospital	0.006	0.011	0.005^***^	0.008
Private Hospital	0.001	0.002	0.001	0.002
Head of Household Characteristics				
Married	0.768	0.794	0.026	0.781
Age (years)	46.67	44.58	−2.09^***^	45.62
Male	0.794	0.742	−0.052^***^	0.768
Muslim	0.718	0.779	0.061^***^	0.749
No education	0.825	0.838	0.013	0.832
Observations	1886	2800		4686

^†^ *p* < 0.1, ^**^ *p* < 0.05, ^***^ *p* < 0.01, ^±^ estimated using student’s T-test, ^¥^this is estimated as the concentration index

Positive improvements are also observed with health indicators. The proportion of household members reporting ill health decreased significantly by approximately 21 percentage points points. In terms of household healthcare-seeking behaviours, the proportion of household members utilising formal public outpatient healthcare services remained largely unchanged. However, a higher proportion of household members utilised informal healthcare services (an increase of approximately 1 percentage points) while utilisation of formal private as well as NGO/missionary healthcare services decreased by approximately 0.4 and 1 percentage points, respectively.

A shift is observed in the distribution of households across Sierra Leone’s four regions (Table 2). The proportion of households living in the Western and Southern regions decreased by approximately 4, and 14 percentage points, respectively. A corresponding increase is observed with the proportions of households living in the worst conflict-affected regions of the Eastern (an increase of approximately 5 percentage points) and Northern regions (an increase of approximately 13 percentage points) [37]. The concentration index is positive for both years (0.06 and 0.068), although it is statistically significant for only 2011 suggesting that catastrophic health expenditure is concentrated more amongst the richer population (Table 2).

### Regression analysis

Table 3 presents the elasticities of a series of linear probability models of the incidence of catastrophic health expenditure. The elasticities represent the magnitude of the associations between household characteristics and the probability of incurring catastrophic health expenditure. Similar to previous studies [9–13], the incidence of catastrophic health expenditure is observed to be correlated with a wide range of household characteristics and healthcare-seeking behaviours. In the pooled data (2003 and 2011), the probability of incurring catastrophic health expenditure increases with household size, the incidence of illness and with the use of formal and informal health care services. For example, a 1 percent increase in household size increases the probability of incurring catastrophic health expenditure by approximately 14 percentage points.

**Table 3:** Linear probability model of catastrophic health expenditure (defined at a threshold of 10%) showing marginal effects

Model Covariates	2003	2011	Difference^±±^	Pooled
Below median expenditure	0.0444	0.00998	−0.0344	0.0261
	(0.028)	(0.019)	(0.037)	(0.017)
Married	0.0773	−0.0211	−0.0984^†^	0.0233
	(0.048)	(0.025)	(0.051)	(0.025)
Age (years)	0.00243	−0.00477	−0.00720	−0.00167
	(0.005)	(0.004)	(0.007)	(0.003)
Age squared	−0.0000192	0.0000526	0.0000718	0.0000238
	(0.000)	(0.000)	(0.000)	(0.000)
Male	−0.0185	0.0144	0.0329	0.00716
	(0.044)	(0.022)	(0.043)	(0.021)
Muslim	0.0330	−0.00354	−0.0366	0.0157
	(0.029)	(0.021)	(0.036)	(0.018)
No education	−0.00278	0.00817	0.0110	0.00304
	(0.040)	(0.025)	(0.041)	(0.024)
Log household size	0.157^***^	0.138^***^	−0.0183	0.142^***^
	(0.024)	(0.018)	(0.031)	(0.015)
Children under 5 years	−0.000669	−0.000710	−0.0000406	−0.000550
	(0.001)	(0.001)	(0.001)	(0.001)
Adults over 65 years	0.00124	0.00120	−0.0000386	0.000899
	(0.001)	(0.001)	(0.002)	(0.001)
Unemployed adults	−0.000492	−0.000110	0.000382	−0.000274
	(0.000)	(0.000)	(0.000)	(0.000)
Ill health	0.00504^***^	0.00329^***^	−0.00175^**^	0.00456^***^
	(0.000)	(0.001)	(0.001)	(0.000)
Rural	0.00642	0.00855	0.00212	0.0115
	(0.030)	(0.024)	(0.045)	(0.019)
Eastern region^±^	−0.154^***^	−0.00530	0.149^**^	−0.0742^**^
	(0.052)	(0.032)	(0.060)	(0.030)
Northern region^±^	−0.147^***^	0.0123	0.160^***^	−0.0650^**^
	(0.054)	(0.031)	(0.059)	(0.031)
Southern region^±^	−0.106^**^	0.126^***^	0.233^***^	−0.00677
	(0.053)	(0.036)	(0.068)	(0.033)
Formal Public	0.00466^***^	0.00729^***^	0.00263^**^	0.00546^***^
	(0.001)	(0.001)	(0.001)	(0.000)
Formal NGO & Missionary	0.00462^***^	0.0108^***^	0.00619^†^	0.00597^***^
	(0.002)	(0.002)	(0.003)	(0.001)
Formal Private	0.00483^***^	0.00643^***^	0.00161	0.00520^***^
	(0.001)	(0.001)	(0.001)	(0.001)
Informal Private	0.00413^***^	0.00364^***^	−0.000481	0.00316^***^
	(0.001)	(0.001)	(0.002)	(0.001)
Public Hospital	0.00607^**^	0.00935^***^	0.00328	0.00825^***^
	(0.003)	(0.002)	(0.003)	(0.002)
Private Hospital	0.00722	−0.00475^**^	−0.0120^**^	−0.00233
	(0.005)	(0.002)	(0.006)	(0.002)
2011 (base category: 2003)	-	-	-	−0.0505^***^
				(0.017)
Constant	−0.0692	0.0131	0.0823	0.0107
	(0.144)	(0.081)	(0.175)	(0.079)
Observations	1886	2800		4686
*R* ^2^	0.305	0.335		0.332
adj. *R* ^2^	0.296	0.329		0.329

^†^ *p* < 0.1, ^**^ *p* < 0.05, ^***^ *p* < 0.01

Standard errors in parentheses

^±^Base category: Western region

^±±^Standard errors estimated using 100 bootstrap replications

Compared to households located in the Western region, households in other regions were more likely to experience financial risks associated with seeking healthcare. For example, the probability of incurring catastrophic health expenditure for households living in the Eastern region is approximately 7 percentage points higher than households in the Western region.

The pooled cross-sections, while providing useful insights into the determinants of catastrophic health expenditure, mask important changes that occurred over time and the resulting implications for households’ exposure to financial risks. A comparison of coefficient estimates between 2003 and 2011 shows that the magnitude and direction of effect vary between the two years (Table 3). For example, in 2003 households located in the Eastern, Southern and Northern regions were less likely to incur catastrophic health expenditure compared to households in the Western region (by approximately 15, 11 and 15 percentage points, respectively). However in 2011, this effect is reversed with households in the other regions more likely to incur catastrophic health expenditure compared to households in the Western region. This is especially true for households located in the Southern region where the probability of incurring catastrophic health expenditure is approximately 13 percentage points higher compared to the Western region.

In both years, the incidence of ill health is associated with a higher probability of incurring catastrophic health expenditure; however, the magnitude of the effect is significantly lower in 2011 (by approximately 0.2 percentage points). Similarly, the use of any healthcare facility is associated with a higher probability of incurring catastrophic health expenditure. However in 2011, the magnitude of this effect is higher for formal public outpatient healthcare services by approximately 0.3 percentage points.

### Oaxaca-blinder decomposition analysis

The Oaxaca-Blinder decomposition analysis (Table 4) quantifies the extent to which changes in mean and coefficients (elasticities) of characteristics described above contributed to the observed decrease in the incidence of catastrophic health expenditure (or CHE gap). The aggregate decomposition (top panel, Table 4) shows that both changes in mean distribution of characteristics (total distributional effect) and to a lesser degree, changes in the impact of these characteristics (total coefficient effect) contributed significantly to the CHE gap.

**Table 4:** Decomposing the change in catastrophic health expenditure at 10% threshold

Aggregate Decomposition						
	Mean					
CHE 2011	32.0^***^					
CHE 2003	50.1^***^					
CHE gap	−18.1^***^					
Contribution	Mean	Percent				
Distributional Effect	−14.0^***^	77%				
Coefficient Effect	−6.65^***^	37%				
Interaction	2.56	−14%				
Detailed Decomposition						
	Distributional Effect		Coefficient Effect		Interaction Effect	
Contribution	Mean	Percent	Mean	Percent	Mean	Percent
Below median expenditure	−0.61	4%	−1.91	29%	0.473	18%
Married	0.194	−1%	−7.56^†^	114%	−0.247	−10%
Age (years)	−0.509	4%	−33.6	505%	1.51	59%
Age squared	0.356	−3%	17.1	−257%	−1.33	−52%
Male	0.0966	−1%	2.61	−39%	−0.172	−7%
Muslim	0.201	−1%	−2.63	40%	−0.222	−9%
No education	−0.004	0%	0.904	−14%	0.014	1%
Unemployed adults	−0.355	3%	1.17	−18%	0.276	11%
Rural area	0.0404	0%	0.129	−2%	0.0134	1%
Region	−1.14^**^	8%	1.25^**^	−19%	−0.427	−17%
Log household size	−0.42	3%	−2.9	44%	0.049	2%
Children under 5 years	0.24	−2%	−0.057	1%	0.0146	1%
Adults over 65 years	−0.067	0%	−0.017	0%	0.0021	0%
Ill health	−10.5^***^	75%	−6.96^**^	105%	3.62^**^	141%
Formal Public	0.234	−2%	2.35^***^	−35%	0.132	5%
Formal NGO & Missionary	−0.384^**^	3%	0.741^**^	−11%	−0.515^**^	−20%
Formal Private	−2.01^***^	14%	1.02	−15%	−0.671	−26%
Informal Private	0.240^†^	−2%	−0.05	1%	−0.028	−1%
Public Hospital	0.314^†^	−2%	0.189	−3%	0.169	7%
Private Hospital	0.0613	0%	−0.131^†^	2%	−0.102	−4%
Constant			21.8	−328%		
Total	−14.0^***^	100%	−6.65^***^	100%	2.56	100%
Observations	4686		4686		4686	

^†^ *p* < 0.1, ^**^ *p* < 0.05, ^***^ *p* < 0.01

Total distributional effect is largely driven by changes in the mean distribution^4^ of ill health (which contributed 75% to the total distributional effect), changes in regional distribution of households (which contributed 8% to the total distributional effect) and changes in the distribution of formal private and NGO/missionary health facilities (which contributed 14% and 3%, respectively to the total distributional effect) (middle panel, Table 4).

**Fig. 1:**
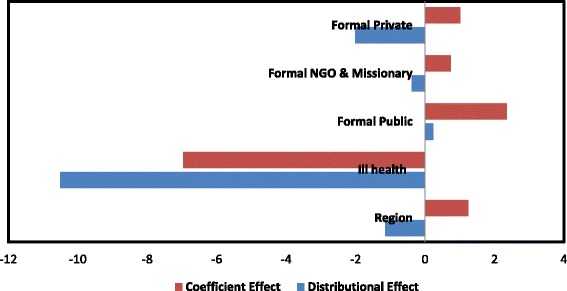
Significant contributors to changes in catastrophic health expenditure between 2003 and 2011

Total coefficient effect is largely driven by changes in the impact of ill health; however, regional variations in the incidence of catastrophic health expenditure as well as changes in the impact of public healthcare service-use have counteracting effects (Fig. 1).

### Robustness check

The robustness of these findings is tested using different thresholds for defining catastrophic health expenditure (Table 5). The CHE gap is observed to be narrower at higher thresholds. The detailed decomposition shows that while the distributional effects are largely robust to the threshold used, the precision of coefficient effect estimates vary with threshold.

**Table 5:** Robustness check- Decomposing 2011–2003 change in catastrophic health expenditure at different thresholds

	5%	10%	15%	20%	25%
2011	43.5^***^	32.0^***^	24.1^***^	17.8^***^	13.4^***^
2003	66.1^***^	50.1^***^	38.5^***^	30.7^***^	24.5^***^
CHE gap	−22.6^***^	−18.1^***^	−14.4^***^	−12.9^***^	−11.1^***^
Distributional Effect	−15.3^***^	−14.0^***^	−10.9^***^	−8.60^***^	−6.11^***^
Coefficient Effect	−9.75^***^	−6.65^***^	−4.98^**^	−4.73^**^	−5.45^***^
Interaction	2.47	2.56	1.48	0.393	0.445
Detailed Decomposition: Distributional Effect					
Below median expenditure	−0.454	−0.61	−0.585	−0.438	−0.253
Married	0.136	0.194	0.288	0.187	0.151
Age (years)	0.247	−0.509	0.412	0.51	0.133
Age squared	−0.0951	0.356	−0.471	−0.252	0.0814
Male	−0.119	0.0966	0.32	0.342	0.186
Muslim	0.0933	0.201	0.0754	0.0157	−0.0726
No education	−0.0405	−0.00354	−0.0333	0.00378	−0.0149
Unemployed adults	−0.348	−0.355	−0.236	−0.42	−0.214
Rural area	−0.328	0.0404	0.0175	0.00533	−0.0432
Region	−0.528	−1.14^**^	−1.36^***^	−2.05^***^	−1.81^***^
Log household size	−0.456	−0.42	−0.267	−0.399	−0.376
Children under 5 years	0.249	0.24	0.182	0.266	0.369
Adults over 65 years	−0.0913	−0.0672	−0.00296	−0.0669	−0.0438
Ill health	−12.7^***^	−10.5^***^	−7.45^***^	−5.08^***^	−3.13^***^
Formal Public	0.133	0.234	0.304	0.31	0.307
Formal NGO & Missionary	−0.281^**^	−0.384^**^	−0.540^***^	−0.481^**^	−0.411^**^
Formal Private	−1.06^***^	−2.01^***^	−2.42^***^	−2.07^***^	−2.08^***^
Informal Private	0.166^†^	0.240^†^	0.336^**^	0.371^**^	0.393^**^
Public Hospital	0.123	0.314^†^	0.411^†^	0.501^**^	0.567^**^
Private Hospital	0.00152	0.0613	0.116	0.143	0.162
Detailed Decomposition: Coefficient Effect					
Below median expenditure	−2.49	−1.91	−3.37^†^	−2.98	−2.34
Married	−4.47	−7.56^†^	−10.5^***^	−9.43^**^	−6.91^†^
Age (years)	−17.5	−33.6	−9.07	1.53	1.35
Age squared	11.7	17.1	3.92	5.74	4.25
Male	−2.97	2.61	6.48^†^	7.20^†^	4.43
Muslim	−0.752	−2.63	−0.746	0.183	1.02
No education	4.61	0.904	1.84	−1.61	0.904
Unemployed adults	−0486	1.17	1.62	2.42^†^	1.7
Rural area	4.97^**^	0.129	1.19	2.22	2.5
Region	0.567	1.25^**^	1.03^**^	1.08^**^	0.986^**^
Log household size	−1.8	−2.9	2.96	−9.90^†^	−14.0^***^
Children under 5 years	1.9	−0.0574	−0.271	0.455	0.701
Adults over 65 years	−0.226	−0.0172	0.76	−0.132	0.449
Ill health	−9.57^***^	−6.96^**^	−1.97	0.897	1.22
Formal Public	3.84^***^	2.35^***^	−0.316	−1.05	−1.48
Formal NGO & Missionary	0.637^**^	0.741^**^	0.405	0.497	0.635^†^
Formal Private	2.73^***^	1.02	−1.13	−1.28	−1.74^†^
Informal Private	0.00682	−0.0502	−0.307	−0.475^**^	−0.587^***^
Public Hospital	0.331^**^	0.189	0.0993	−0.072	−0.112
Private Hospital	−0.0407	−0.131^†^	−0.183^**^	−0.199^**^	−0.214^**^
Constant	−0.724	21.8	2.59	0.174	1.77
Observations	4686	4686	4686	4686	4686

^†^ *p* < 0.1, ^**^ *p* < 0.05, ^***^ *p* < 0.01

## Discussion

Following the end of the brutal civil conflict in Sierra Leone, the health sector underwent a series of reforms aimed at strengthening both demand- and supply-side functions of the health system. These include the devolution of administrative responsibilities and funds to local councils in 2004; the reintroduction of the cost-recovery scheme and user fees in public health facilities in 2006; the Free Health Care Initiative (FHCI) in 2010 and the accompanying human resources for health (HRH) reformsand widespread reconstruction and refurbishment of public healthcare facilities nationwide [25–29]. Covering a time period over which these health reforms were implemented, this study provides insights into the extent to which changes in household healthcare-seeking behaviours, influenced by ongoing health reforms and wider economic recovery contributed to changes in households’ vulnerability to healthcare financial risks. This study finds that the incidence of catastrophic health expenditure decreased significantly between 2003 and 2011. Using an Oaxaca-Blinder decomposition approach, we show that the observed decrease (or CHE gap) represents a net effect resulting from changes in the mean distribution and impact of a range of determinants having both contributory and/or counteracting effects.

Changes in both the distribution and coefficient effect of ill health between 2003 and 2011 made the largest contribution to the CHE gap. Between 2003 and 2011, the proportion of household members reporting ill health decreased significantly, contributing to a reduction in the incidence of catastrophic health expenditure. Similarly, the impact of ill health on the likelihood of incurring catastrophic health expenditure reduced significantly between the two study years. In addition to severe disruptions to livelihood, conflicts have been linked to outbreaks of diseases, particularly infectious disease due to disruptions in safe drinking water sources and sanitation [38]. The conflict in Sierra Leone was no exception, and was characterized by a widespread destruction of lives and livelihood, the collapse of public infrastructure and the displacement of millions of people [20, 21]. As peace and security returned to Sierra Leone, public infrastructure were rebuilt and household livelihoods re-established [23]. The distributional and coefficient effects possibly reflect these wider improvements in household living and environmental conditions, resulting in better health outcomes, reduced severity of diseases and lower healthcare costs as a consequence.

The distributional effect of household regional location provides further indication of general improvements in household economies in the post-conflict period. The distributional effect of regional location, capturing the shift in populations from the least conflict-affected regions (the Western and Southern regions) to the worst affected regions (the Northern and Eastern regions) is indicative of regional economic growth as peace and security returned [37, 39]. This in turn is likely to have improved household economic conditions and households’ ability to cope with the associated cost of seeking healthcare services.

By contrast the regional coefficient effect, capturing regional variations in exposure to financial risk suggests that these positive changes may not have been felt equally across all regions. Compared to households living in the Western region, those in other regions were less likely to incur catastrophic health expenditure in 2003 but more likely in 2011. Changing population distribution and accessibility of health infrastructure is likely to explain this effect. For example, only 16 health facilities were functioning at the height of the conflict in 1996, with the majority of these located in the Western Region [40]. Therefore unavailability of functioning healthcare facilities in the Eastern, Southern and Northern regions would have resulted in households forgoing healthcare services in 2003. By 2011, progress had been made in rebuilding and refurbishing health infrastructure including higher level facilities and lower level peripheral health units [41]. This would have resulted in better access and use of healthcare services across all regions. However, long-standing socioeconomic variations across regions [39, 42–44] may have limited the ability of households, who seek healthcare in poorer regions to cope with the costs of accessing health care services compared to households in the wealthier Western region.

The distributional and coefficient effect of facility-type provides interesting insights into the evolution of health service provision in the post-conflict era. A statistically significant decrease in the use of private facilities including NGO and missionary-owned facilities is observed between 2003 and 2011 while an increase is observed in the use of public health facilities (although not statistically significant). In the aftermath of the conflict, NGOs and private actors played an important role in the provision of healthcare [45], filling the gap left by the destruction of public health infrastructure during the conflict. However, in the period covered by this study (2003 to 2011), progress had been made in rebuilding and refurbishing public health facilities [23], thus leading to an increase in the supply and use of these services.

The decrease in the use of formal private and NGO healthcare services significantly reduced households’ exposure to financial risk. However, those who continued to use NGO-owned health facilities faced a higher risk of incurring catastrophic health expenditure in 2011 (captured in the coefficient effect). An increase in prices of healthcare services between 2003 and 2011 is likely to explain the observed coefficient effect of NGO- and privately-owned healthcare facilities. NGO-owned facilities in Sierra Leone largely operate on a similar basis to privately owned facilities, charging patients a fee-for-service at the point of care [30, 46]. Although not directly affected by the cost-recovery policy, the introduction of user fees in public health facilities in 2006 may have had an indirect effect on prices in private and NGO-owned facilities. In other settings, prices in the private health sector have been shown to be responsive to user fee changes within the public health sector. For example, the introduction of user fees in public facilities in Cambodia was associated with an increase in prices in private facilities [47] while the removal of user fees in public facilities in Uganda was associated with a decrease in prices within private health facilities [48].

The distributional effect of public health services suggests an increase in households’ exposure to the financial risk of seeking healthcare. While this effect was not found to be statistically significant, the coefficient effect provides more insight into the implication of public health facility-use on households’ exposure to financial risk. In both study years, the risk of incurring catastrophic health expenditure increased with the proportion of household members using public health services. However, this effect is significantly higher in 2011 suggesting higher financial risks faced by households. This effect may partly be explained by health financing reforms including the reintroduction of the cost recovery scheme and user fees in public health facilities in 2006 which would have resulted in higher out-of-pocket health expenditure for those utilizing these services in 2011 [2, 30, 31]. Furthermore, an increase in the range of service options available in public health facilities as they were rebuilt and refurbished, is likely to have resulted in an increase in the quantity of services demanded per visit, higher out-of-pocket payments and catastrophic health expenditure, as a consequence. Although through the FHCI, exemption rules and fee waivers apply for vulnerable populations using public health facilities, there is limited evidence on the effectiveness of these policies in protecting households from the financial burden of healthcare. For example, less than complete implementation and shortfalls in medicines and supplies have limited the impact of the FHCI for recent mothers and children under five years [49–52].

These findings are in part, sensitive to the catastrophic health expenditure threshold. The CHE gap varies between 11 and 23%, depending on the threshold used for defining catastrophic health expenditure. Nevertheless, the proportionate contribution of the total coefficient and distributional effects to the CHE gap at each threshold are comparable. The detailed distributional effects appear robust to thresholds, while the coefficient effect varies in some cases. For example, the coefficient effect of ill health and public health facility-use is significant only at lower thresholds suggesting equal effects of these determinants in both years at higher thresholds.

### Study limitations

The robustness of our findings could be affected by the extent of measurement errors in household expenditure data. The precision of household expenditure estimates from survey data is subject to recall periods over which data are collected and the differences in how goods were itemised between the two study years [53]. Given that the 2003 and 2011 SLIHS differ in terms of recall periods and the goods itemised, this is likely to have implications for both estimates of total household expenditure and estimates of the incidence of catastrophic expenditure. For example, longer recall periods over which food and frequently purchased non-food expenditure were collected (3–12 month in 2003 vs. 5 days in 2011) may have resulted in an underestimation of total household expenditure in 2003. Conversely, longer recall periods over which expenditure on inpatient healthcare services and medicines were collected (4 weeks in 2011 vs. 2 weeks in 2003) may have resulted in an underestimation of total health expenditure in 2011. Although we mitigate potential bias due to differences in the degree to which goods were itemized by including only goods and services for which data were available in both years, we cannot rule out potential bias arising from variations in recall periods.

Furthermore, differences in the recall periods over which health expenditure and other household expenditure were collected may have affected our estimates of the incidence of catastrophic health expenditure. Households are likely to recall and accurately report health expenditure occurring over a shorter recall period [53, 54]. On the other hand, expenditure on other household expenditure collected over a longer recall period is likely to have been underestimated [53, 54]. This may have resulted in an overestimation of our estimates of the incidence of catastrophic health expenditure.

The findings of this study illustrate an important limitation of this measure of catastrophic health expenditure. Catastrophic health expenditure is conditional on healthcare use and captures the financial risks associated with accessing care. This means that the measure of catastrophic health expenditure defined in this study captures only financial risks for those who seek health care but fails to account for those who do not seek health care when ill due to an inability to pay. Although we find some evidence that regional socioeconomic inequality may have accounted for higher catastrophic health expenditure in 2011, estimates of the concentration index in 2011 suggests that in the total population, catastrophic health expenditure was concentrated amongst those who potentially have the ability to pay for health care when needed. The situations where health care is not sought due to an inability to pay or when health care services are not available may be equally impoverishing – for example an unresolved health problem could prevent adults in a household from working, thus compromising household living standards. The catastrophic health expenditure measure defined in this study does not detect these and is an inevitably partial measure to understanding the links between health, health seeking behavior and impoverishment.

## Conclusion

The findings of this study suggest that while efforts have been made to address supply-side constraints to accessing healthcare in the years following the conflict in Sierra Leone [55], financial risks faced by households persist. This has important policy implications particularly in settings transitioning out of conflict. At the end of the conflict, efforts of the Sierra Leone government and international donor agencies were directed towards rebuilding health infrastructure and strengthening supply-side functions of the health system through policies aimed at increasing the supply of health workers. However, significant financial barriers to accessing public health services remained and continued to worsen the risk of household impoverishment through ill health. For example, the reintroduction of the cost recovery scheme in 2006 without consideration of households’ ability to pay for these services would have compromised households’ recovery in the aftermath of the conflict. Our results suggest that households located in the poorer regions most affected by the conflict were more likely to have experienced an increase of financial risk associated with healthcare use.

While rebuilding and refurbishing destroyed health infrastructure (supply-side initiatives) is a crucial element to improving access to quality healthcare services in the aftermath of conflict, for policy-makers, this study highlights the importance of complementary demand-side mechanisms aimed at protecting households, at a time when households are themselves recovering from severe losses. These demand-side mechanisms including health insurance schemes and partial or full health care fee waivers (voucher schemes and health equity funds) have been shown to reduce out-of-pocket payments amongst the most vulnerable populations in other settings [56]. Although Sierra Leone is making efforts towards implementing more equitable health care financing reforms, for example through the FHCI and the proposed national health insurance scheme, the FHCI has been shown to provide partial financial protection for vulnerable populations due to implementation challenges [50].

## Abbreviations

CHE: Catastrophic Health Expenditure
FHCI: Free Health Care Initiative
HRH: Human Resources for Health
NGO: Non-governmental Organization
SLIHS: Sierra Leone Integrated Household Survey.

## Footnotes

1 The recall period differed by type of healthcare service utilised

2 At a rate of 1USD = 4349.20 Leones

3 Data on visits to patent medicine vendors was collected in 2011 only and is therefore not included in informal health service-use.

4 Only statistically significant contributions are discussed. These are depicted graphically in Fig 1.
